# Significantly Increased Risk of Cardiovascular Disease among Patients with Gallstone Disease: A Population-Based Cohort Study

**DOI:** 10.1371/journal.pone.0076448

**Published:** 2013-10-03

**Authors:** Muideen Tunbosun Olaiya, Hung-Yi Chiou, Jiann-Shing Jeng, Li-Ming Lien, Fang-I Hsieh

**Affiliations:** 1 School of Public Health, Taipei Medical University, Taipei, Taiwan; 2 The Stroke Center and Department of Neurology, National Taiwan University Hospital, Taipei, Taiwan; 3 Department of Neurology, Shin Kong WHS Memorial Hospital, Taipei, Taiwan; Scuola Superiore Sant'Anna, Italy

## Abstract

**Objective:**

To investigate whether gallstone disease (GD) increases the risk of developing cardiovascular disease (CVD) in a large population-based cohort.

**Methods:**

A study population including 6,981 patients with GD was identified from The Taiwan National Health Insurance Research Database between 2004 and 2005. GD patients were defined as patients with principal discharge diagnoses of cholelithiasis using the ICD-9-CM code 574. 27,924 patients without GD were randomly selected and matched for age and gender. All patients were followed for 6 years or until diagnosis for CVD. Cox proportional hazards regression model was used to assess the risk of developing CVD with adjustment for age, gender and co-morbid conditions.

**Results:**

During the six years follow-up period, 935 patients with GD and 2,758 patients without GD developed CVD. Patients with GD had an elevated risk of CVD (HR, 1.32; 95% CI, 1.22-1.43) when compared with those without GD. Similar relationship was observed when CVD was categorized i.e. stroke (HR, 1.15; 95% CI, 1.01-1.32), coronary heart disease (HR, 1.42; 95% CI, 1.28-1.58) and heart failure (HR, 1.31; 95% CI, 1.00-1.73). When GD was classified according to the level of severity, using patients without GD as reference, the risks of CVD were elevated in patients with non-severe GD (HR, 1.34; 95% CI, 1.24-1.46) as well as those with severe GD (HR, 1.20, 95% CI, 1.02-1.40), after adjusting for age, gender and comorbidities. In age-stratified analysis, patients aged 18-40 years with GD were at higher risk of developing CVD (HR, 1.42; 95% CI, 1.09-1.84) than older GD patients.

**Conclusion:**

This study found an increased risk of CVD in patients diagnosed with GD. The excess risk was particularly high in younger GD patients. Prevention of GD could help reduce the risk of developing CVD, and the better effect could be achieved for the younger age groups.

## Introduction

Gallstone disease (GD) and cardiovascular disease (CVD) are both common public health problems, representing a major economic burden [1-4]. Once considered as the disease of the West [2], GD is becoming increasingly prevalent in some other developed countries of the world [1]. In the United States, GD is the leading cause of hospital admissions related to gastrointestinal problems [2], with about 800,000 cholecystectomies performed annually [5]. Similarly, the current epidemiological transition in low- and middle-economic countries has pushed the global burden of CVD higher [3,6]; hence CVD currently accounts for 30% of global deaths and will continue to lead global mortality trend in the future [7]. Most GD are asymptomatic [8], however, in symptomatic cases, symptoms may be mild such as biliary pain (colic) or severe such as acute cholecystitis, cholangitis, pancreatitis, gallbladder cancer, obstruction of the bowel and complications of cholecystectomy [9,10].

Although a causal association between GD and CVD remains unclear, previous studies have attributed the association between these conditions to be a result of shared features in the pathophysiological mechanism [11-13] i.e. cholesterol accumulation in the gallbladder [14], as well as arteriosclerosis in the arterial wall [15]. Moreover, diseases/conditions directly related to excessive cholesterol such as hypercholesterolemia and hyperlipidemia as well as other conditions such as hypertension, diabetes mellitus, peripheral vascular disease, alcoholism and chronic obstructive pulmonary disease are well known risk factors of CVD [16-19]. Hence, the persistent identification of some of these CVD-associated conditions in GD [13,20,21] suggests possible association.

To the best of our knowledge, this is the first study to comprehensively explore the association between these 2 conditions i.e. the association between GD and CVD was examined exhaustively for different categories of both conditions. For instance, GD was further defined based on level of severity. Moreover, our study provided explicit information on the relationship between GD and specific CVDs such as stroke and heart failure. Therefore, using a large population-based dataset obtained from the National Health Insurance (NHI) in Taiwan, we aimed to comprehensively investigate whether GD increases the risk of CVD based on a cohort study design.

## Methods

### Ethical considerations

The National Health insurance Research Database (NHIRD) is a secondary database. The information on the identity of subjects from the database was scrambled before it was released for research purpose. The privacy and confidentiality of all beneficiaries were safeguarded by the Taiwan National Health Research Institute (NHRI). In this study, ethics approval was provided by the Institutional Review Board of Taipei Medical University.

### Data Sources

In Taiwan, the National Health Insurance (NHI) Program was inaugurated in 1995, and as at 2007, this program has enrolled about 22.6 million out of 22.96 million of Taiwan’s population [22]. This accounts for about 98.5% of the population in Taiwan. The NHIRD contains data for inpatient claims of 1 million subjects representing about 5% of NHI beneficiaries. We obtained a longitudinal data from January 1, 2000 to December 31, 2010, consisting of patient’s medical history and clinical features at presentation. According to a recent validation study, the Taiwan NHIRD was found to be a valid and accurate resource for population-based research [23].

### Study cohort

Using the 2000-2010 records of ambulatory care expenditures by visits and inpatient expenditures by admissions, patients with an index case of GD between January 1, 2004 and December 31, 2005 were identified. GD cases were patients with principal discharge diagnoses of cholelithiasis using the International Classification of Diseases, Ninth Revision, Clinical Modification (ICD-9-CM) code 574 [24]. GD was categorized according to the severity of the disease i.e. non-severe GD and severe GD. Non-severe GD was defined as ICD-9-CM codes 574 (without 577.0 or 577.1 or 576.1) or 574.1 or 574.2 or 574.4 or 574.5 or 574.7 or 574.9. Severe GD was defined as gallstone-associated complications i.e. acute cholecystitis (ICD-9-CM codes 574.0 or 574.3 or 574.6 or 574.8), biliary pancreatitis (ICD-9-CM codes 574 plus 577.0 or 577.1), and acute cholangitis (ICD-9-CM codes 574 plus 576.1), as well as gallstone-associated surgical and endoscopic procedures i.e. non-elective cholecystectomy due to cholecystitis (ICD-9-CM codes 574.0 or 574.3 or 574.6 or 574.8, plus 51.22 or 51.23) and gallstone cases receiving endoscopic retrograde cholangiopancreatography-ERCP (ICD-9-CM codes 574 plus 51.10 or 51.11 or 51.64 or 51.84 or 51.85 or 51.86 or 51.87 or 51.88 or 52.13). See Table S1 for the frequency of GD in the study cohort.

We excluded patients with entry age of <18 and >80 years, patients who have been diagnosed with CVD at baseline, and those with unknown identification number, date of birth and sex. Moreover to reduce the possibility of misdiagnosis, patients with a history of primary and secondary liver cancer, cancer of the gallbladder and extra-hepatic bile ducts, pancreatic cancer, gastric cancer, malignant neoplasm of other sites, hematological malignancy, biliary pancreatitis by alcohol use, as well as patients with immunodeficiency syndrome or human immunodeficiency virus infections (HIV or HIV/AIDS) were also excluded.

Using the same exclusion criteria, a comparison group was created from the remaining patients in the dataset. The comparison group was made up of patients with no case of GD between January 1, 2000 and December 31, 2005. These were randomly selected and frequency-matched with GD group at a ratio of 1:4 according to 5-year age interval and gender. The index date of entry into this study for the GD group was defined as the date the patients presented with the first case of GD in the claims data. For the non-GD group, the index date was the date the patients were first identified in the claims data between January 1, 2004 and December 31, 2005. The study cohort was followed for 6 years for a diagnosis of CVD while withdrawal from the insurance program was censored.

### Data collection

Based on the ICD-9-CM codes, CVD was defined as stroke (ICD-9-CM codes 430-437, excluding 432), coronary heart disease (ICD-9-CM codes 410 or 411 or 413 or 414) and heart failure (ICD-9-CM code 428.0). If multiple incident cases of CVD were diagnosed in the same subject, only the first diagnosed case during follow-up was included. All the first diagnosed CVD cases were further categorized as stroke, CHD, and heart failure. The claims data was searched for information on age and gender as well as co-morbid conditions such as peripheral vascular disease, diabetes mellitus, hyperlipidemia, hypertension, chronic obstructive pulmonary disease (COPD), alcoholism, chronic liver diseases and hereditary or acquired hemolytic anemia. Co-morbidities were also defined using the ICD-9-CM codes (see Table S2).

### Statistical analysis

To compare the baseline characteristics between GD group and non-GD group, continuous variables were summarized as means and standard deviations, and categorical variables as frequencies and percentages. Statistical comparison of categorical variables was performed using Pearson’s Chi-square test while means of continuous variables were compared using Student’s t-test. The incidence densities of CVD for the 2 groups were estimated by dividing the number of incident CVD cases by person-years at risk. Kaplan-Meier survival analysis was carried out to compare the CVD event free probability between the groups within 6-year follow-up period.

Univariate and multivariate survival analysis with Cox proportional hazard regression model was used to estimate crude and adjusted hazard ratios (HRs) of CVD and 95% confidence interval (CI) among patients with GD compared to those without GD for the time of follow-up. The Cox regression model was verified to satisfy the proportional hazard assumption. For the multivariate-adjusted model, we simultaneously included age, sex and all the co-morbid variables as covariates.

The association between GD and CVD was estimated for the different categories of GD and CVD. To further minimize the potential confounding effect of important CVD risk factors such as hypertension, diabetes mellitus and age, we performed analyses restricted to the following patients: those with no history of hypertension at baseline; those with no history of diabetes mellitus at baseline; those with no history of both hypertension and diabetes mellitus at baseline; those with no history of hypertension, diabetes mellitus and entry age of <40 years at baseline; and finally those with no comorbidity (i.e. without peripheral vascular disease, chronic obstructive pulmonary disease, hypertension, hyperlipidemia, alcoholism, chronic liver disease, and anemia) and entry age of <40 years at baseline. Statistical significance was set at P<0.05 and all analyses were conducted using SAS software version 9.3 (SAS Institute, Cary, NC).

## Results

**Figure 1:**
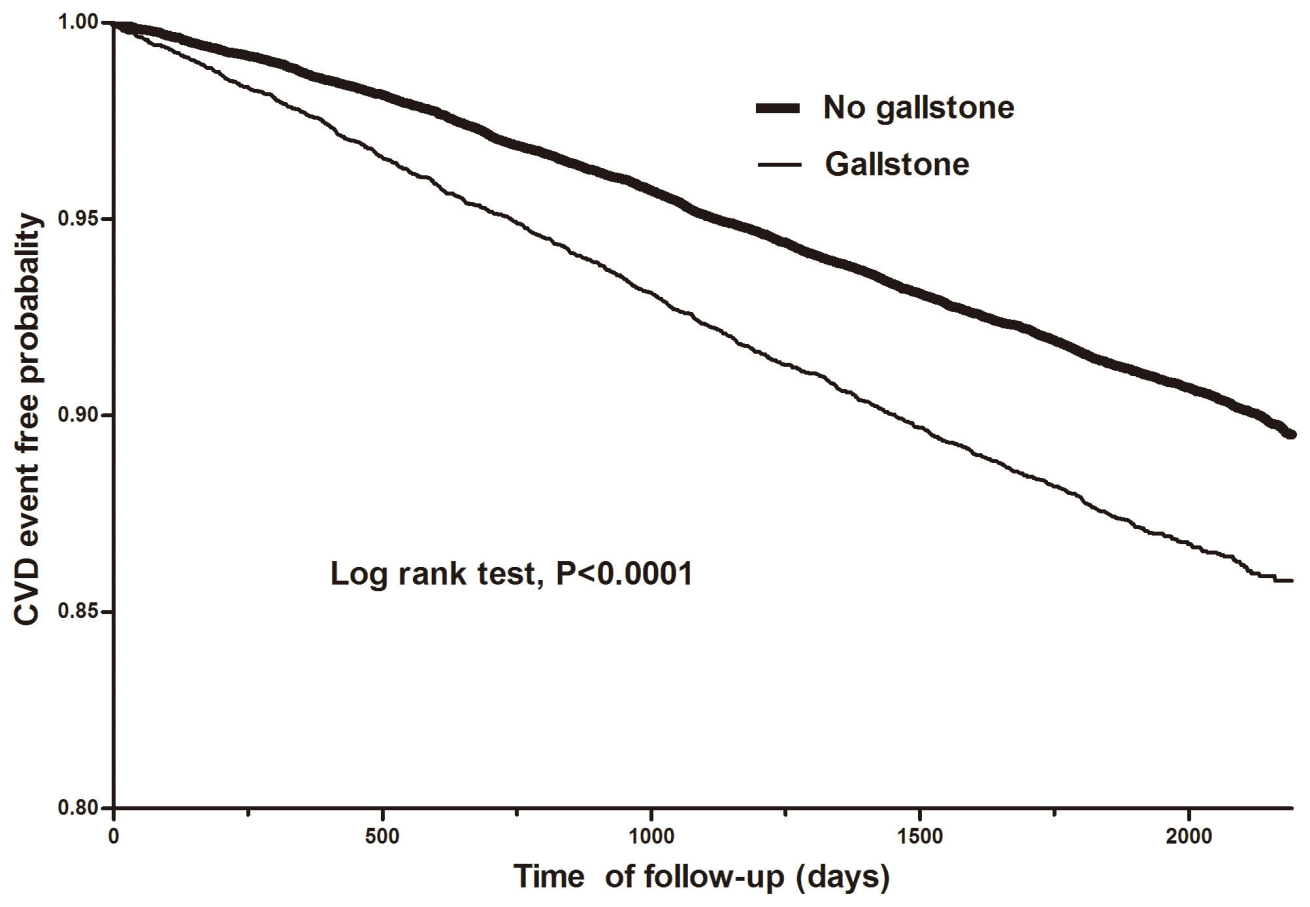
Kaplan-Meier 6-year CVD event free probability curves for gallstone and non-gallstone disease groups.

A total of 34,905 patients were eligible for the study, of which 6,981 were diagnosed with GD at baseline, while 27,294 had no case of GD. Since the two groups were matched according to gender and age, there was no significant difference in age and gender distributions between the two groups (Table 1). Patients with GD had a higher prevalence of all the measured comorbidities and all were found to be statistically significant except peripheral vascular disease. Figure 1 shows the Kaplan-Meier curve estimating the CVD event free rates between the two groups over 6 years follow-up period. Overall, there was a significant difference in the CVD event free curves between the GD and non-GD groups (log-rank test: p<0.0001).

**Table 1:** Baseline characteristics of patients by gallstone disease status.

**Characteristic**	**Gallstone Disease**			***P* value**
	**No (n=27924)**		**Yes (n=6981)**	
	**n (%)**		**n (%)**	
Gender				1.00
Male	12228 (43.8)		3057 (43.8)	
Female	15696 (56.2)		3924 (56.2)	
Age (years)				
18-30	3068 (11.0)		767 (11.0)	1.00
31-40	5512 (19.7)		1378 (19.7)	
41-50	7416 (26.6)		1854 (26.6)	
51-60	6135 (22.0)		1533 (22.0)	
>60	5793 (20.8)		1449 (20.8)	
Peripheral vascular disease				0.21
Yes	200 (0.7)	60 (0.9)		
No	27724 (99.3)	6921 (99.1)		
Chronic Obstructive Pulmonary Disease				<0.0001
Yes	3663 (13.1)	1359 (19.5)		
No	24261 (86.9)	5622 (80.5)		
Diabetes mellitus				<0.0001
Yes	2219 (8.0)	917 (13.1)		
No	25705 (92.0)	6064 (86.9)		
Hyperlipidemia				<0.0001
Yes	2878 (10.3)	1211 (17.4)		
No	25046 (89.7)	5770 (82.6)		
Hypertension				<0.0001
Yes	4672 (16.7)	1526 (21.9)		
No	23252 (83.3)	5455 (78.1)		
Alcoholism				<0.0001
Yes	59 (0.2)	51 (0.7)		
No	27865 (99.8)	6930 (99.3)		
Chronic Liver Disease				<0.0001
Yes	3402 (12.2)	2463 (35.3)		
No	24522 (87.2%)	4518 (64.7)		
Anemia				0.008
Yes	59 (0.2)	27 (0.4)		
No	27865 (99.8)	6954 (99.6)		

During the 6 years of follow-up, 935 patients with GD were diagnosed with CVD. Patients with GD diagnosis had a 1.32-fold increased risk of CVD (95% CI, 1.22-1.43) compared to those without GD diagnosis (Table 2). When GD was stratified according to severity and using patients without GD as reference, the risk of CVD was elevated and similar for both non-severe and severe GD groups. However, in the multivariate adjusted model, the risk of CVD was more elevated in the non-severe GD group (HR, 1.34; 95% CI, 1.24-1.46) than the severe GD group (HR, 1.20; 95% CI, 1.02-1.40). CVD risk was also increased in the two categories of severe GD when compared to the non-GD group, although the risk was slightly more elevated in patients with gallstone-associated procedures (HR, 1.32; 95% CI, 1.07-1.62) compared to those with gallstone-associated complications (HR, 1.23; 95% CI, 1.04-1.45).

**Table 2:** Incidence density and risk of cardiovascular disease by gallstone disease status.

	**No CVD**	**CVD**	**Incidence ^a^**	**Crude HR (95% CI)^b^**	**Adjusted HR (95% CI)^c^**
No Gallstone Disease (reference)	25166	2758	18.1	1.00	1.00
Gallstone Disease (Total)	6046	935	26.0	1.45 (1.35-1.56)***	1.32 (1.22-1.43)***
Non-Severe Gallstone Disease	4922	759	26.0	1.45 (1.34-1.57)***	1.34 (1.24-1.46)***
Severe Gallstone Disease	1124	176	26.3	1.47 (1.26-1.71)***	1.20 (1.02-1.40)*
Gallstone-associated complications ^d^	1009	155	25.9	1.45 (1.23-1.70)***	1.23 (1.04-1.45)*
Gallstone-associated procedures ^e^	480	97	33.2	1.85 (1.52-2.27)***	1.32 (1.07-1.62)**

Abbreviation: CVD, cardiovascular disease; CI, confidence interval; HR, hazard ratio.

a Per 1000 person years.

b Within 6 years of follow up.

c Adjusted for age, gender, peripheral vascular disease, chronic obstructive pulmonary disease, diabetes mellitus, hyperlipidemia, hypertension, alcoholism, chronic liver disease, and anemia.

d Gallstone-associated complications include acute cholecystitis, biliary pancreatitis, acute cholangitis.

e Gallstone-associated procedures include non-elective cholecystectomy and endoscopic retrograde cholangiopancreatography

* *P*<0.05; **P<0.01; ***P<0.001

The multivariate analysis demonstrated a significant association between GD and all the categories of CVD i.e. stroke (HR, 1.15; 95% CI, 1.01-1.32), coronary heart disease (HR, 1.46; 95% CI, 1.28-1.58) and heart failure (HR, 1.31; 95% CI, 1.00-1.73), after controlling for age, gender and selected co-morbidities (Table 3). The multivariate regression model also indicated that all the co-morbid conditions except hemolytic anemia were independent risk factors for CVD among the GD group. The results of the subgroup analyses confirmed the association between GD and CVD (Table 4). For instance, the risk of CVD still remained elevated in GD patients with no comorbidity and age <40 years at baseline (HR, 1.77; 95% CI, 1.28-2.45).

**Table 3:** Multivariate Cox regression analysis for cardiovascular disease.

**Characteristic**	**CVD**		**Stroke**	**CHD**		**Heart failure**
	**HR (95% CI)**		**HR (95% CI)**	**HR (95% CI)**		**HR (95% CI)**
GD	1.32 (1.22-1.43)***		1.15 (1.01-1.32)*	1.42 (1.28-1.58)***		1.31 (1.00-1.73)^#^
Male gender	1.08 (1.01-1.15)*		1.36 (1.22-1.51)***	0.95 (0.87-1.04)		0.81 (0.64-1.02)
Age (in years)^a^						
31-40	2.33 (1.76-3.09)***		2.71 (1.56-4.72)***	2.35 (1.65-3.34)***		1.30 (0.46-3.69)
41-50	4.45 (3.41-5.81)***		4.68 (2.76-7.93)***	4.55 (3.27-6.34)***		3.20 (1.26-8.11)*
51-60	7.54 (5.79-9.81)***		9.48 (5.64-15.94)***	7.06 (5.08-9.82)***		5.31 (2.12-13.30)***
>60	11.52 (8.85-15.0)***		19.97 (11.92-33.45)***	7.71 (5.53-10.74)***		15.14 (6.14-37.28)***
PVD	1.33 (1.03-1.73)*		1.54 (1.05-2.28) *	1.21 (0.82-1.76)		1.52 (0.67-3.41)
COPD	1.18 (1.09-1.28)***		1.01 (0.88-1.15)	1.26 (1.13-1.40)***		1.60 (1.25-2.06)***
Diabetes mellitus	1.17 (1.07-1.28)***		1.43 (1.24-1.65)***	0.98 (0.85-1.11)		1.08 (0.79-1.48)
Hyperlipidemia	1.21 (1.11-1.31)***		1.03 (0.89-1.20)	1.43 (1.27-1.60)***		0.77 (0.56-1.06)
Hypertension	1.65 (1.53-1.78)***		1.70 (1.51-1.93)***	1.55 (1.39-1.72)***		2.06 (1.61-2.65)***
Alcoholism	2.10 (1.36-3.23)***		2.02 (0.96-4.26)	1.73 (0.92-3.22)		7.13 (2.62-19.42)***
CLD	1.10 (1.01-1.19) *		1.01 (0.88-1.16)	1.20 (1.08-1.34)**		0.91 (0.67-1.22)
Anemia	1.10 (0.55-2.19)		1.30 (0.42-4.04)	1.12 (0.47-2.69)		-

Abbreviations: GD, gallstone disease; CVD, cardiovascular disease; CHD, coronary heart disease; PVD, peripheral vascular disease; COPD, chronic obstructive pulmonary disease; CLD, chronic liver disease; HR, hazard ratio; CI, confidence interval.

a 18-30 years as reference

^#^ *P*=0.05; *P<0.05; **P<0.01; ***P<0.001

**Table 4:** Subgroup analysis for cardiovascular disease risk in patients with gallstone disease compared with those with no gallstone disease.

	**No hypertension at baseline**		**No diabetes at baseline**		**No hypertension and diabetes at baseline**		**No hypertension, no diabetes and age <40 years at baseline**		**No co-morbidity** ^a^ **and age <40 years at baseline**	
	No GD	GD	No GD	GD	No GD	GD	No GD	GD	No GD	GD
No CVD	21509	4923	23407	5347	20618	4562	8075	1914	6997	1257
CVD	1743	532	2298	717	1576	455	201	73	160	47
Crude HR ^a^(95% CI)	1.00	1.38 (1.25-1.52) ***	1.00	1.42 (1.30-1.54) ***	1.00	1.36 (1.23-1.51) ***	1.00	1.60 (1.22-2.09) ***	1.00	1.72 (1.24-2.38) **
Adjusted HR (95% CI)	1.00	1.28 ^b^ (1.16-1.42)***	1.00	1.32^c^ (1.21-1.44) ***	1.00	1.29 ^d^(1.16-1.44) ***	1.00	1.49 ^d^(1.13-1.98) **	1.00	1.77^e^ (1.28-2.45) ***

Abbreviation: CVD, cardiovascular disease; GD, gallstone disease; CI, confidence interval; HR, hazard ratio.

a No co-morbidity means without peripheral vascular disease, chronic obstructive pulmonary disease, hypertension, hyperlipidemia, alcoholism, chronic liver disease, and anemia

b Adjusted for age, gender, peripheral vascular disease, chronic obstructive pulmonary disease, diabetes mellitus, hyperlipidemia, alcoholism, chronic liver disease, and anemia.

c Adjusted for age, gender, peripheral vascular disease, chronic obstructive pulmonary disease, hypertension, hyperlipidemia, alcoholism, chronic liver disease, and anemia.

d Adjusted for age, gender, peripheral vascular disease, chronic obstructive pulmonary disease, hyperlipidemia, alcoholism, chronic liver disease, and anemia.

e Adjusted for gender.

* P<0.05; **P<0.01; ***P<0.001

The risk of CVD was significantly higher in the GD group compared to the non-GD group for both males (HR, 1.29; 95% CI, 1.15-1.44) and females (HR, 1.35; 95% CI, 1.22-1.50) (Table 5). When the patients were stratified into age groups, patients aged 18-40 years with GD demonstrated the highest risk of developing CVD with HR of 1.42 (95% CI, 1.09-1.84), compared to HR of 1.35 (95% CI, 1.21-1.51) for patients aged 41-60 years and HR of 1.24 (95% CI, 1.10-1.39) for patients >60 years.

**Table 5:** Incidence density and risk of cardiovascular disease for gallstone and non-gallstone disease groups stratified by age and sex.

	**No gallstone**			**Gallstone**				
	**CVD**		**Incidence ^a^**	**CVD**		**Incidence ^a^**	**Crude HR (95% CI)^b^**	**Adjusted HR (95% CI)**
	**No**	**Yes**		**No**	**Yes**			
Gender								
Female	14203	1493	17.3	3414	510	25.3	1.47 (1.33-1.63)***	1.35 (1.22-1.50)*** ^c^
Male	10963	1265	19.0	2632	425	27.0	1.43 (1.28-1.59)***	1.29 (1.15-1.44)*** ^c^
Age (in years)								
18-40	8352	228	4.7	2057	88	7.6	1.64 (1.28-2.10)***	1.42 (1.09-1.84)** ^d^
41-60	12278	1273	17.2	2923	464	26.7	1.57 (1.41-1.75)***	1.35 (1.21-1.51) *** ^d^
>60	4536	1257	42.4	1066	383	55.2	1.32 (1.17-1.47)***	1.24 (1.10-1.39) *** ^d^

Abbreviation: CVD, cardiovascular disease; CI, confidence interval; HR, hazard ratio.

a Per 1000 person years.

b Within 6 years of follow up.

c Adjusted for age, peripheral vascular disease, chronic obstructive pulmonary disease, diabetes mellitus, hyperlipidemia, hypertension, alcoholism, chronic liver disease, and anemia.

d Adjusted for age, gender, peripheral vascular disease, chronic obstructive pulmonary disease, diabetes mellitus, hyperlipidemia, hypertension, alcoholism, chronic liver disease, and anemia.

* *P*<0.05; **P<0.01; ***P<0.001

## Discussion

In the present study, GD patients showed an increased prevalence of CVD risk factors such as hypertension, diabetes mellitus, hyperlipidemia and chronic obstructive pulmonary disease. This finding is consistent with a previous study conducted among patients who underwent cholecystectomy for GD in Mexico [13]. This study found a 1.32-fold increased risk of CVD in patients with GD after adjusting for potential confounding factors. Other studies have also reported an association between GD and CVD [12,25,26]. However, most of these studies were unable to clearly explain a temporal relationship between these two conditions.

The association between GD and CVD may be due to shared pathophysiological features. The precipitation of excess cholesterol in bile as solid crystals is required for cholesterol gallstone formation [14,27]. Similarly, the pathogenesis of CVD is characterized by a complex series of events leading to cholesterol accumulation and eventually the formation of atherosclerotic plaque [15,28]. Another possible explanation for this association may be shared metabolic pathway. A report by Twickler et al. suggests that low plasma levels of insulin-like growth factor-1 (IGF-1) may lead to the development of both GD and coronary heart disease (CHD) [29]. Patients with a low level of growth hormones such as IGF-1 have been reported to be susceptible to altered postprandial gallbladder emptying [30]. This results in prolonged nucleation of monohydrate cholesterol crystals from the supersaturated bile to form macroscopic stones [31]. Similarly, IGF-1 has been reported to be atheroprotective [32,33]. Hence, low levels of this growth hormone could cause apoptosis and vascular dysfunction, a situation which could result in several cardiovascular pathologies like atherothrombosis, myocardial infarction and heart failure [34].

Inflammation could also play an important role in the association between these two conditions. The pathogenesis of GD is characterized by a high level of oxidative stress and inflammation in the gall bladder mucosa [35]. Similarly, atherosclerosis is characterized by inflammation triggered by oxidative stress [36]. Abnormal (high) levels of plasma total homocysteine (tHcy), a prooxidant, have been implicated in these two pathological processes [37,38]. Moreover, increasing concentrations of tHcy has been associated with an increasing level of pro-inflammatory C-reactive protein [39]. Compared to non-GD group, the risk of developing CVD was similar in the non-severe GD group to that in the severe GD group. This finding agreed with that of a recent U.S. study based on mortality data [12], which found no significant difference in the risk of death due to CVD between individuals with GD and those with a more severe condition i.e. those who underwent cholecystectomy for GD. In a separate analysis of the different categories of CVD, GD patients showed an increased risk of all the categories of CVD examined. This finding is consistent with that of a similar retrospective study of the Framingham cohort [26], as our results also showed a significantly increased risk of both coronary heart disease and heart failure in patients with GD.

The association between GD and CVD has been suggested to be due to shared risk factors [13,21]. Owing to the high prevalence of important CVD risk factors in the GD patients as compared to the non-GD patients, the results of our subgroup analyses in which we excluded patients with important CVD risk factors and age >40 years at baseline confirmed the validity of our results. With the subgroup analyses, it is reasonable to conclude that the increased risk of CVD observed in GD patients was more likely to be due to the effect of their GD status since the possible confounding effect of CVD risk factors has been significantly minimized. Moreover, the higher excess risk observed in the younger age groups when compared to older patients (>60 years) may be due to the absence or low prevalence of CVD-associated comorbidities in the younger patients. This finding, coupled with the results of the subgroup analyses affirms the possible causal association between GD and CVD. It also suggested that GD is an important risk factor for premature CVD.

The major limitation of this study is the inadequacies embedded in the use of an administrative based data. Although we adjusted for some important confounding factors associated with CVD, we could not obtain information on other potential covariates like body mass index (BMI), family history as well as lifestyle factors like diet and physical activity intensity. However, we adjusted for COPD as proxy for cigarette smoking in the regression model. COPD is primarily caused by smoking; hence the presence of COPD may independently elevate the risk of CVD [40]. BMI is highly correlated with classical risk factors of CVD such as hyperlipidemia, hypertension and diabetes [41]. Therefore, the adjustment for these factors in the regression model could partially account for the confounding effect of BMI. However, it could not completely rule out the residual confounding effect from BMI and this may undermine the estimates of the study. The possibility of including patients with undiagnosed/asymptomatic GD in the non-GD group could lead to misclassification, thereby underestimating the risk of developing CVD due to GD. However, this would not have biased the conclusions from our findings. There is also the possibility of surveillance bias as patients in the GD group are more likely to be screened for CVD compared to the comparison group. However, because of the high accessibility and affordability of health care services in Taiwan, this could not have compromised the findings of this study.

The strengths of our study should also be stated. Firstly, the estimates of this study are based on a large nationally representative sample. Therefore, the statistical power (nearly 99%) was sufficient for our findings. Secondly, cohort study design was employed in this study, thereby making it possible to ascertain a temporal relationship.

## Conclusion

Patients with GD have an increased risk of developing CVD. The excess risk was particularly high in younger GD patients. Prevention of GD could help reduce the risk of developing CVD, and the better effect could be achieved for the younger age groups.

## Supporting Information

Table S1 **Definition and frequency of gallstone disease in the study cohort.**
(DOC)

Table S2 **Definition of co-morbidities based on the ICD-9-CM codes.**
(DOC)
