# Supplementary material for: Significantly Increased Risk of Cardiovascular Disease among Patients with Gallstone Disease: A Population-Based Cohort Study
Source: PLoS One. 2013 Oct 3;8(10):e76448. doi: 10.1371/journal.pone.0076448 (PMC3789705; doi:10.1371/journal.pone.0076448)
Supplement: Table S1 — Definition and frequency of gallstone disease in the study cohort. (DOC) [file pone.0076448.s001.doc]

**Table S1**. Definition and frequency of gallstone disease in the study cohort.

| **Categories** | **ICD-9-CM codes** | **Frequency (%)** |
| --- | --- | --- |
| **Gallstone disease** |  | **6981** |
| **Non-severe Gallstone Disease** | 574 (without 577.0 or 577.1 or 576.1) or 574.1or 574.2 or 574.4 or 574.5 or 574.7or 574.9 | **5681 (81.4)** |
| **Severe Gallstone Disease** |  | **1300 (18.6)** |
| **Gallstone-related complications** |  | **1164 (16.7)** |
| Acute cholecystitis | 574.0 or 574.3 or 574.6 or 574.8 | 963 (13.8) |
| Biliary pancreatitis | 574 plus 577.0 or 577.1 | 148 (2.1) |
| Acute cholangitis | 574 plus 576.1 | 100 (1.4) |
| **Gallstone-related procedures** |  | **577 (8.3)** |
| Non-elective cholecystectomy | 574.0 or 574.3 or 574.6 or 574.8 plus 51.22 or 51.23 | 290 (4.2) |
| Gallstones receiving ERCP | 574 plus 51.10 or 51.11 or 51.64 or 51.84 or 51.85 or 51.86 or 51.87  or 51.88, 52.13 | 323 (4.6) |
|  |  |  |
|  |  |  |
