# Supplementary material for: Significantly Increased Risk of Cardiovascular Disease among Patients with Gallstone Disease: A Population-Based Cohort Study
Source: PLoS One. 2013 Oct 3;8(10):e76448. doi: 10.1371/journal.pone.0076448 (PMC3789705; doi:10.1371/journal.pone.0076448)
Supplement: Table S2 — Definition of co-morbidities based on the ICD-9-CM codes. (DOC) [file pone.0076448.s002.doc]

**Table S2**. Definition of co-morbidities based on the ICD-9-CM codes.

| **Disease** | **ICD-9-CM codes** |
| --- | --- |
| Peripheral vascular disease | 093.0, 437.3, 440, 441, 443.1, 443.2, 443.8, 443.9, 447.1, 557.1, 557.9, V434 |
| Diabetes mellitus | 250 |
| Hyperlipidemia | 272.0, 272.1, 272.2, 272.3, 272.4 |
| Hypertension | 401, 402, 403, 404, 405 |
| Chronic obstructive pulmonary disease | 416.8, 416.9, 490, 491, 492, 493, 494, 495, 496, 500, 501, 502, 503,504, 505, 506.4, 508.1, 508.8 |
| Chronic liver disease | 571 |
| Anemia | 282, 283 |
| Alcoholism | 303.0, 303.9 |
